# Ovarian Aging: Role of Pituitary-Ovarian Axis Hormones and ncRNAs in Regulating Ovarian Mitochondrial Activity

**DOI:** 10.3389/fendo.2021.791071

**Published:** 2021-12-16

**Authors:** Marco Colella, Danila Cuomo, Teresa Peluso, Ilaria Falanga, Massimo Mallardo, Mario De Felice, Concetta Ambrosino

**Affiliations:** ^1^ Biogem, Istituto di Biologia e Genetica Molecolare, Ariano Irpino, Italy; ^2^ Department of Science and Technology, University of Sannio, Benevento, Italy; ^3^ Laboratory of Pre-Clinical and Translational Research, IRCCS, Referral Cancer Center of Basilicata, Rionero in Vulture, Italy; ^4^ Department of Molecular and Cellular Medicine, College of Medicine, Texas A&M University, College Station, TX, United States; ^5^ Department of Molecular Medicine and Medical Biotechnologies, University of Naples “Federico II”, Naples, Italy; ^6^ Istituto per l’ endocrinologia e l’oncologia “Gaetano Salvatore” (IEOS)-Centro Nazionale delle Ricerche (CNR), Naples, Italy

**Keywords:** ncRNA, mitochondria, ovarian aging, estrogens, FSH (follicle stimulating hormone), LncRNA - long noncoding RNA, miRNA - microRNA, MitomiRs

## Abstract

The number of mitochondria in the oocyte along with their functions (e.g., energy production, scavenger activity) decline with age progression. Such multifaceted functions support several processes during oocyte maturation, ranging from energy supply to synthesis of the steroid hormones. Hence, it is hardly surprising that their impairment has been reported in both physiological and premature ovarian aging, wherein they are crucial players in the apoptotic processes that arise in aged ovaries. In any form, ovarian aging implies the progressive damage of the mitochondrial structure and activities as regards to ovarian germ and somatic cells. The imbalance in the circulating hormones and peptides (e.g., gonadotropins, estrogens, AMH, activins, and inhibins), active along the pituitary-ovarian axis, represents the biochemical sign of ovarian aging. Despite the progress accomplished in determining the key role of the mitochondria in preserving ovarian follicular number and health, their modulation by the hormonal signalling pathways involved in ovarian aging has been poorly and randomly explored. Yet characterizing this mechanism is pivotal to molecularly define the implication of mitochondrial dysfunction in physiological and premature ovarian aging, respectively. However, it is fairly difficult considering that the pathways associated with ovarian aging might affect mitochondria directly or by altering the activity, stability and localization of proteins controlling mitochondrial dynamics and functions, either unbalancing other cellular mediators, released by the mitochondria, such as non-coding RNAs (ncRNAs). We will focus on the mitochondrial ncRNAs (i.e., mitomiRs and mtlncRNAs), that retranslocate from the mitochondria to the nucleus, as active players in aging and describe their role in the nuclear-mitochondrial crosstalk and its modulation by the pituitary-ovarian hormone dependent pathways. In this review, we will illustrate mitochondria as targets of the signaling pathways dependent on hormones and peptides active along the pituitary/ovarian axis and as transducers, with a particular focus on the molecules retrieved in the mitochondria, mainly ncRNAs. Given their regulatory function in cellular activities we propose them as potential diagnostic markers and/or therapeutic targets.

## Introduction

The physiological and the premature ovarian aging are often asymptomatic. Both include the decline in the number of the follicles. The number of follicles is established in early life and they mature later in life. Genetic and environmental factors co-contribute to the establishment of the ovarian reserve (OR) and its following decrease, which is promoted by different mechanisms. The imbalance of the hormonal asset, required for the preservation and maturation of the ovarian follicles, is among them and it can lastly lead to their premature aging and/or death (1). Ovarian follicle maturation is a complex process, defined folliculogenesis, organized in different phases to generate a species dependent number of mature oocytes. The oocyte maturation equally involves changes in the ovarian somatic cells, such as Granulosa Cells (GCs) and Theca Cells (TCs), surrounding the oocytes and supporting its maturation. These cells also will undergo an highly differentiation process involving multiple paracrine interactions, important for the maintenance of normal follicular development (2–4).

The decline in the number of follicles throughout the life is known as follicular atresia, consisting in the apoptosis of the oocytes and of the surrounding follicular cells (5). It is not surprising that mitochondria play a central role in follicular atresia, since they are central executors of the apoptosis (6–10). Recently, it has been shown that mitochondrial biogenesis is among the pathways determining the size of the follicular pool which, as said, is established in early life (11, 12). Furthermore, mitochondria are among the determinants of the cytoplasmic quality of the oocytes that, with nuclear integrity, defines the oocyte quality and competence. Indeed, transfer of mitochondria to oocytes prevents their apoptosis (13, 14). Quality of mitochondria in GCs influences the oocyte since these cells supply them with energy substrates (15).

Mitochondrial quantity is generally reduced in physiological aging, decreasing their ability to supply adenosine triphosphate (ATP) along with their mitochondrial reactive oxygen species (ROS)-scavenger activities (16–23). These concepts are widely accepted however the recent genome-wide association studies conducted to identify genomic loci that influence age at menopause do not evidence its association with gene codifying mitochondrial proteins (24). This finding suggests that mitochondrial dysfunction described in ovarian aging might be primarily due to environmental factors. In fact, diet and chemical toxins, capable of reducing OR, impact on processes leading to follicular atrophy such as control of the oxidative stress (OS), inflammation, hormone secretion and, finally, modulation of the cellular content of the non-coding RNAs (ncRNAs) involved in ovarian aging (25–27). These latter modulate ovarian lifespan and health-span in vertebrates, including mammals, regulating signaling pathways playing pivotal role in follicular atresia as PI3K-AKT, mTOR and estrogen signaling pathways, among the others (28, 29).

Here, we will discuss the literature on the role of pituitary-ovarian hormones in regulating ovarian mitochondrial activity and dynamics associated with ovarian aging. Furthermore, we will discuss the role of ncRNAs as executors of such activities pointing out the role of mitochondrial associated miRNAs (mitomiRNAs, mitolncRNAs).

## Mitochondrial Dysfunction in the Ovarian Aging

Mitochondria are unique organelles in animal cells since they contain a genome (mtDNA) and the enzymes to duplicate and transcribe it. Since DNA-repairing enzymes are not present in mitochondria, mutations are frequently found in mtDNA. They affects mtDNA replication and transcription and increase with aging (30). Impairment in mitochondrial number and function in oocytes and ovarian somatic cells has been associated with ovarian aging and infertility (31). The increase of mitochondrial number characterizes the oogenesis and stops when oocytes are fully mature. In fact, the number of mitochondria is approximately ten-hundred in the human Primordial Germ Cells (PGCs) and becomes about several hundred-thousand in the mature oocyte (32). This suggests that mitochondria could be targets of the hormonal asset controlling this process, as we will discuss later. Since they do not further replicate during the post-fertilization cleavage process, they are distributed amongst the dividing cells and their number has been proposed as a biomarker of embryo viability (33–35). Mitochondrial dysfunction plays a major role in ovarian aging since this organelle contributes to the energetic needs of the oocyte and protect DNA with its ROS-scavenger activity (36). The ROS are produced in the cells as a result of their metabolic activity, including ATP production. Their increase induces a cascade of events at the level of the mitochondrion that, if left unchecked, will promote mtDNA, mutations and accumulation of misfolded proteins (UPRmt), causing cellular damage and death (37–39). Noteworthy, mitochondrial dysfunction promotes an increased oxidative stress that, in turns, impairs the energy production required to sustain the metabolic needs during oogenesis and follicle maturation (40).

The ability of ovarian cells to neutralize the OS diminishes with age, resulting in a steady decline in oocyte quality (41–43). In humans, increased production of ROS within the ovarian cells correlate with follicular atresia, poor oocyte quality and infertility (44). The aged GCs exhibit an increase of OS that promotes their apoptosis and senescence. Indeed, OS induces shortening of telomeres, a process described in ovarian aging (45, 46). Several studies have been conducted in mice to investigate how changes in the redox state can damage the oocytes and GCs. Gene expression profiling of aged oocytes revealed a down-regulation of mRNAs codifying mitochondrial antioxidant proteins such as peroxiredoxin 3 (PRDX3) and thioredoxin 2 (TXN2), in addition to cytosolic antioxidant proteins (47–49). Their imbalance affects directly mitochondrial activity, including the ovarian steroidogenesis as shown in SOD-2 deficient mice, due to an impairment in the cholesterol transport to mitochondria following the reduction of mitochondrial content of STAR protein (50). The reduction of these “protective proteins” makes the oocytes far more susceptible to damage induced by changes in the oxidative state.

Moreover, the damage of mitochondrial dynamics has been described in women affected from premature and physiological ovarian aging. As we will discuss here, this might result from the imbalance of the hormones active along the pituitary-ovarian axis (51).

## Mitochondrion-Nucleus Crosstalk: A Focus on ncRNAs Acting as Anterograde and Retrograde Signals

**Figure 1:**
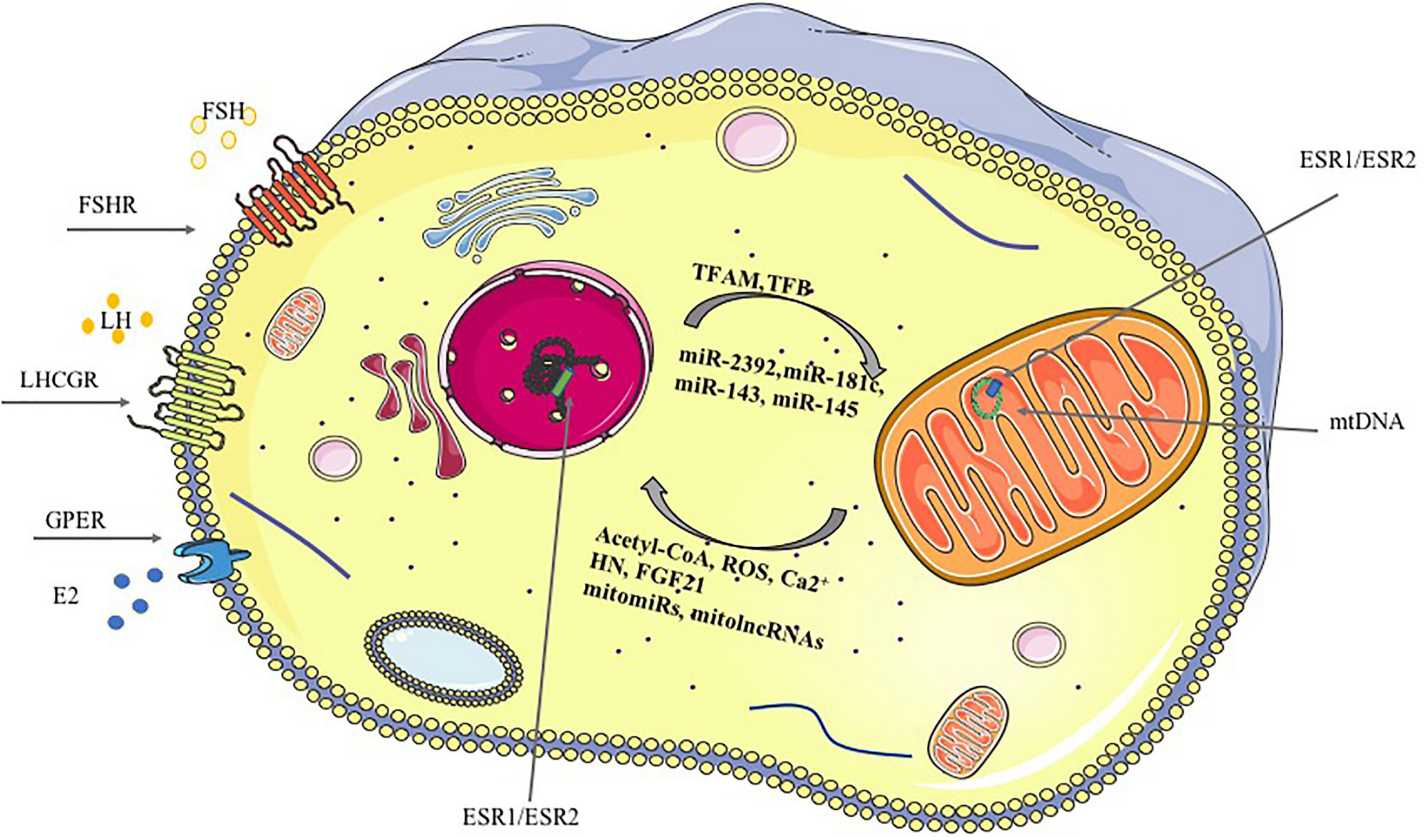
Mitochondria-nucleus crosstalk, anterograde and retrograde signaling. Gonadotropins and ovarian hormones signaling pathways regulating mitochondrial dynamics and function are reported in the figure underlining the cellular comportment involved. Anterograde and retrograde signals are reported for the mitochondrion-nucleus crosstalk. Anterograde signals (nucleus to mitochondria) include: the nuclear-encoded transcription factors (TFs, TFAM and TFB that bind the mtDNA, nuclear receptors (NRs) and ncRNAs, among the others. Retrograde signals (mitochondria-nucleus) include: Ca2+, ROS, mitomiRs, mitolncRNAs, among the others (53).

An extensive bidirectional communication network between mitochondria and the nucleus has been characterized (52). The anterograde (nucleus to- mitochondria) signals have been extensively described whereas more recently the retrograde (mitochondria-to-nucleus) pathway components, including Acetyl coenzyme A (Acetyl-CoA), ROS and ncRNAs, have been described (**Figure 1**). The retrograde signals, ROS and intracellular calcium (Ca^2+^) increase or/and reduction of Acetyl-CoA or ATP, disarrange the activity of several cellular signaling pathways impairing the cellular content of Nuclear Respiratory Factor 1 (NRF1), a protein regulating the mitochondrial transcription factor A (*TFAM*) and, finally, the mitochondrial gene transcription (54, 55). Furthermore, proteins secreted by the mitochondria under stress condition, defined mitokines, might act as retrograde signals (56). Humanin (HN) and fibroblast growth factor 21 (FGF21) are among them (57, 58). Interestingly, mitochondrial HN has been recognized as a protective factor for GCs (59). Indeed, its inactivation through gene targeting modified ovarian organization and promoted the apoptosis of GCs. Such effects were confirmed *in vitro* in granulosa tumor cell line (KGN) which were more viable, when treated with exogenous humanin. Concordantly, its level in follicular fluids positively correlates with the ovarian reserve (60).

Non-coding RNAs control numerous cellular functions by modulating specific signaling pathways, also involved in ovarian aging (26). Several classes of ncRNAs may impact directly and/or indirectly on mitochondrial biology (61). They are considered mediators of the anterograde and retrograde mitochondrion-nucleus crosstalk, controlling several cellular activities at transcriptional and post-transcriptional levels. Mitochondria-localized miRNAs and lncRNAs, termed mitomiRs and mt-lncRNAs respectively, directly regulate mitochondrial gene expression. Their uptake by the mitochondria as well their transcription from mtDNA has been suggested (62). Certainly, it is still ambiguous whether mitochondrial ncRNAs (mt-ncRNAs) are transcribed inside the mitochondria or the nuclei, as result of mitochondrial genes integrated into the nuclear genome (63). The former suggestion is not entirely surprising since mitochondria contain genomes and can produce mitochondrial-specific nucleic acids and proteins. Some studies have indicated the presence of RNA interference components in the mitochondria implying their functional importance (64). MitomiRs are short single stranded RNA molecules with a unique size ~17-25, with no 5’ cap, short 3’ overhangs, and with specific thermodynamic features as opposed to canonical miRNAs. It has been speculated that some of these characteristics might facilitate their entry into mitochondria. However, it is still challenging to accurately characterize the mechanisms determining their localization because of the technical difficulties in separating isolated and uncontaminated organelles.

However, mt-ncRNAs are among the molecules used by mitochondria to communicate with the nucleus to ensure cellular homeostasis since they control rapid cell stress responses, energy balance and apoptosis controlling the cellular content of proteins both at transcriptional and translational levels (65, 66). Although mt-ncRNAs have not been investigated in ovary, we will discuss them here because they modulate mitochondrial processes involved in ovarian aging, such as abundance, metabolism, and scavenger activity (**Table 1**). For instance, miR-2392, together with Argonaute 2 (*Ago2*), has been reported to partially inhibit mtDNA transcription in tongue squamous cell carcinoma cells (TSCC), resulting in down-regulation of oxidative phosphorylation and up-regulation of glycolysis (67). At the translational level, it has been shown that miR-181c in concert with Ago2 can repress the translation of the mitochondrial cyclooxygenase (*Cox1*) mRNA, determining a remodeling of complex IV in cardiac dysfunction (68, 69, 78, 79). Noteworthy, *Cox1* transcript is among the transcripts induced during oocyte maturation (80). Similarly, miR-378 can inhibit the mitochondrial expression of Atp6 (71) that modulates mouse follicle development and oocyte maturation as well as aromatase levels (70). Furthermore, miR-378 modulates Cyp450 in non-human primates ovaries (72). Furthermore, miR-378 is included in a network of miRNAs regulating follicular atresia. On the other hand, mitomiRs can enhance mitochondrial translation (81, 82). For example, same miRNA has been suggested to mobilize *ND1* and *Cox1* mRNAs to mitochondrial ribosomes through Ago2. Furthermore, the growth arrest specific 5 (GAS5) a lncRNA diminished in physiological and environmentally promoted ovarian aging, has been reported as mt-lncRNA and involved in the modulation of mitochondrial tricarboxylic acid flux (26, 83). The results above summarized suggest the role of the mitomiRs in ovarian aging although the mode of action remains largely unexplored and enigmatic.

**Table 1:** miRNAs and lncRNAs and their targets for mitochondrial function in ovarian aging.

miRNAs	Target	Tissue	Model/Cell lines	Reference
miR-2392	mtDNA/AGO2	Oral and maxillofacual region	TSCC	(67)
miR-181c	Cox1/GRP78	Heart/Ovary	*In vivo* Rats/A2780, SKOV3 and human tissues	(68, 69)
miR-378	Aromatase, Atp6, Cyp450	Ovary, Heart, Ovary	Porcine ovaries, HL-1, Cynomolgus macaques	(70–72)
miR-143	KRAS	Ovary	Granulosa cells/ovaries from mouse	(73)
miR-145	ARL5B	Ovary	SKOV3	(74)
**lncRNAs**				
lncND5, lncND6, lncCYT b	ND5, ND6 and Cyt b	Ovary	Granulosa cells (mouse)	(66)
MDL1,MDL1AS	mitoepigenetic processes	Ovary	Germinal Vesicle Oocytes	(75)
GAS5	mTOR	Ovary	Ovaries from mouse	(76)
lncH19	STAT3	Ovary	KGN	(77)

Additionally, cytoplasmic miRNAs impact a variety of mitochondrial processes, including energy metabolism, fusion and fission. The present work origins from our belief that the impairment of some ncRNAs (miR-143, miR-145, GAS5, etc.) could damage mitochondrial function and dynamics accelerating the ovarian aging (76). For instance, miR-145, shown to be overexpressed in ovaries from middle aged mice as well as in follicular fluids from Diminished Ovarian Reserve (DOR) patients, has been associated with poor mitochondrial function in ovarian cancer cells marked by decreased mtDNA copy numbers, ATP production, membrane potential and, finally, the expression levels of mitochondrial markers (74). *In vivo* and *in vitro* studies showed that follicular stimulating hormone (FSH) significantly decreased miR-143 expression, found deregulated in ovarian aging by our and other studies. It has been shown that miR-143 inhibits estradiol production, and proliferation of GCs regulating the cell cycle-related genes expression by targeting KRAS (73). Lastly, it has been reported that its increase promoted mitochondrial damage in myocardial infarction (84). Unfortunately, this has not been verified in the ovarian follicles.

Additionally, lncRNAs have been reported as modulators of mitochondrial function (66, 79). They are ncRNAs, long more than 200 nucleotides, and have a characteristic structure containing domains for RNA, protein and possibly DNA binding. Their structural flexibility and plasticity contribute to the regulation of several cellular processes. LncRNAs have been detected in different cell compartments, including mitochondria (79). Their biological activities depend on their localization in the cell and comprise transcriptional regulation, post-transcriptional activity, metabolic function, and chromosome remodeling. Increasing evidence suggests that lncRNAs can be encoded by nuclear and mitochondrial DNA and some of them have been outlined to regulate mitochondrial biology directly or indirectly (61). The ones retrieved in mitochondria are known as mt-lncRNAs and might work as retrograde signals. For instance, a sense ncRNA (SncmtRNA) and two antisense ncRNAs (ASncmtRNas-1, -2) can be transported in the nucleus from the mitochondria and act as mediator of the crosstalk between these organelles. The mitochondrial transcripts *ND5*, *ND6*, and *Cyt b* are target of three mt-lncRNAs, *lncND5*, *lncND6*, and *lncCyt b*. These last are complementary to their respective mRNAs and the duplex formation increases the mRNAs stability (66, 85). Since ND5, ND6, and Cyt b are components of the NADH-deidrogenase, it is easily conceivable the role that these mt-lncRNAs might have in establishing mitochondrial activity and dynamics. Only one paper explored their function in reproductive tissue using *in vitro* models. Experiments performed *in vitro* with culture of GCs in medium supplemented or not with FSH, showed that FSH induced mitochondrial activity, verified by evaluation of membrane potential, ATP content, and of mt-ND6 proteins (86).

Other mt-lncRNAs have been identified as Long Intergenic noncoding RNA predicting CARdiac remodelling (LIPCAR), Mitochondrial D-Loop 1 (MDL1) and Mitochondrial D-Loop 1 antisense (MDL1AS) in tissues other than ovaries (87). The last two have been suggested as executor of mitoepigenetic processes whose dynamic nature has been shown in oocytes and GCs (75). It is well established that mitochondria import various nuclear-encoded lncRNAs. An example is SRA1, a steroid receptor RNA activator, that is downregulated in cumulus cells from old females and upregulated in steroid hormone-responsive tumors, including ovarian cancers. It can recruit co-repressors modulating nuclear receptors activity because of the interaction with SLIRP (SRA stem-loop interacting RNA-binding protein) (88, 89). Interestingly, *Slirp*-knockout females are sub-fertile whereas males have defects in sperm motility and mitochondrial morphology (88, 89). Another example is the RNA component of the RNA processing endoribonuclease (RMRP) transported by RNA binding proteins such as GRSF1 localized in RNA granules. In spite of the fact that it has not been investigated in follicle maturation and/or aging, it has been shown increased in platin-resistant ovarian cancers (90). In other tissues, it is described as p53 inhibitor (91). Finally, some lncRNAs can affect indirectly mitochondrial biology by modulating mitochondrial key pathways. For instance, GAS5 can induce growth-arrest after inhibition of mTOR, known to regulate mitochondrial function. This suggests its potential ability to impact mTOR activity in the mitochondria. In our study, previously mentioned, we showed downregulation of GAS5 in ovaries from middle aged mice suggesting yet again that mitochondrial dysfunction can promote ovarian aging which is consistent with the derangement of the AKT pathway prediction by the IPA analysis (76). Noteworthy as reported above, GAS5 has been also described as mt-lncRNA that alters tricarboxylic acid cycle disrupting metabolic enzyme tandem association of fumarate hydratase, malate dehydrogenase and citrate synthase (83).

## Pituitary Gonadotropins Modulate Ovarian Mitochondrial Morphology, Dynamics, and Steroidogenic Activity

As discussed above, mitochondria play a central role in follicular atresia. It is well established that the oocyte relies largely on mitochondria to acquire competence for fertilization (92, 93). Additionally, early embryonic development depends on their number and quality in the oocytes (94, 95). Furthermore, mitochondria control proliferation, differentiation and activity of GCs along with their apoptosis (96). Therefore, mitochondria might be regarded as targets of the signaling pathways determining the follicle number, quality and surviving, which are adversely affected by aging (51). Pituitary gonadotropins are survival factors for the ovarian follicular cells. Their imbalance characterizes the physiological and premature ovarian aging because they activate survival pathways in the follicle that will determine its growth or death. Although mitochondria are key compartments in this decision, the role of pituitary gonadotropins and of their intracellular mediators in controlling mitochondrial dynamics and activity is largely undefined in ovarian aging.

Follicle stimulating hormone (FSH) is the most efficient gonadotropin in preventing follicular atresia and GCs apoptosis under different conditions, including hypoxic or oxidative stress exposure. Few studies have addressed if FSH could modulate mitochondrial activity and biogenesis through ncRNAs. For instance, it has been reported that FSH modulates the expression of several miRNAs in the ovaries (97). These miRNAs modulate mitochondrial ROS-scavenger activity in other tissues and cell types. An example is miR-143 which has been shown to inhibit mitochondrial activity in human colon cancer cells (HCT116). Particularly, the superoxide dismutase 1 (SOD1) was downregulated by stable expression of miR-143 that increased the ROS production in cells treated with oxaliplatin (98). Noteworthy, miR-143 is negatively regulated by FSH and its increase has been associated with ovarian aging. Taken together, the data suggest that its increase in aged ovaries translates into a less efficient ROS-scavenger activity. No other papers have directly addressed this point, neither characterized other miRNAs nor lncRNAs regulated by FSH and involved in such processes.

Some reports have described the mitochondria as direct targets of FSH under stress conditions. In particular, under hypoxic conditions FSH stimulates the mitochondrial biogenesis in GCs by a process suggestive of its involvement in the degradation of the damaged organelles as well as of their regeneration (99). This finding is in agreement with previously published results obtained in GCs exposed to H_2_O_2_ to induce oxidative stress (100). FSH has a major role in preserving mitochondrial integrity under stress conditions in ovary and it is defined as an active factor in reducing mitophagy induced by oxidative stress and in preventing the death of GCs. Such an effect results from the inhibition of the PINK1-Parkin pathway activated under oxidative stress conditions. Indeed, FSH suppresses Pink1, promotes the dissociation of Parkin from mitochondria and, consequently, it increases the resistance of the GCs to oxidative stress. Noteworthy, Pink1 translation is limited by lncH19 resulting in a reduced mitophagy (101). The reduction of lncH19 expression in GCs results in inhibition of proliferation and increased apoptosis of GCs (77).

GCs are the major site of synthesis of steroid hormones that is strictly controlled by FSH. Mitochondria play a critical role in steroid synthesis and FSH modulates this mitochondrial activity (102, 103). Such aspect has been investigated and molecularly characterized in rat primary GCs showing that prohibitin (PHB) was a FSH responsive transcript. The mitochondrial increase of PHB levels correlated with the stimulation of the steroidogenesis, specifically of the estrogens and progesterone. Furthermore, it has been shown that FSH stimulation promoted PHB phosphorylation by the activation of the ERK1/2 pathway (104). PHB also mediated the pro-survival activity of the FSH in GCs regulating the expression of the anti-apoptotic genes (105). Lastly, PHB maintained the mitochondrial morphology, particularly of the cristae by modulating the processing of mitochondrial dynamin like GTPase (OPA1) (106, 107). This role in preserving mitochondrial fusion and cristae morphogenesis has been firstly evidenced in mouse embryonic fibroblasts (MEFs) and, then, in rat immature GCs overexpressing PHB (108–110). Again, the evaluation of the role of the PHB-OPA1 axis in this model points to mitophagy as a target of the FSH signaling pathway. Finally, other reports showed the role of mTOR pathway in FSH-modulated mitophagy during follicular development. The reported data evidenced that FSH treatment increased the levels of hypoxia-inducible factor 1-alpha (HIF-1α) promoting the autophagy. Interestingly, FSH induced autophagy correlated with an incomplete mitophagy process. Indeed, the Authors reported that the FSH-mediated autophagy increased the mitochondrial membrane potential that was abolished in presence of the autophagy inhibitors due to the PINK1-Parkin pathway (111).

Reduced levels of the luteinizing hormone (LH) characterize both physiological and premature ovarian aging (112, 113). Although suggested since 1975, LH role in modulating mitochondrial function and dynamics has been poorly investigated in ovarian aging. At that time, Robinson and co-authors observed that LH, administrated subcutaneously, increased the rates of cholesterol translocation and the rates of ovarian mitochondrial cholesterol side-chain cleavage in immature rats (114). Recently, more insights have been provided in a study assessing the effects of LH in cultured GCs from infertile women having low (L), medium (M), and high (H) LH serum levels during ovarian stimulation. The bioinformatic analyses of the data suggested an impairment of the mitochondrial activity, involving the mitochondrial dehydrogenase, associated with both insufficient (L) and excessive (H) recombinant LH (rLH) treatment. Morphologically GCs from the L and H groups had an increased number of autophagosomes. Furthermore, swollen and rounder mitochondria were detected with excessive rLH whereas reduced levels of LH promoted the appearance of forked mitochondria (115). Another study specifically addressed the point of the regulation of mitochondrial dynamics by LH in the luteal cells. Plewes and co-authors demonstrated that Dynamin-Related Protein 1 (DRP1), a key mediator of mitochondrial fission, is regulated by LH *via* PKA (116). The LH stimulation decreased the association of DRP1 with the mitochondria indicating that DRP1 was required for optimal LH-induced progesterone biosynthesis. Taken together, these findings place DRP1 as an important target downstream of PKA in steroidogenic luteal cells. LH also is a modulator of the retrograde signaling since it modulates the expression of humanin in luteal cells (117). Although luteal cells do not seem to have a role in ovarian aging, this mechanism has been here discussed since they derive from GCs and TCs. However, it is has been shown that LH acts directly on GCs (118–120).

The discussed data suggest that mitochondria are targets of the main pituitary gonadotropins. FSH-mediated mitophagy, *via* activation of different pathways, is an important mechanism of FSH mediated follicular growth and development other than survival. However, the relationship between FSH, mitophagy and apoptosis remains elusive in GCs and more studies are needed to evaluate the role of FSH-mediated mitochondrial integrity, mitophagic flux and the survival of fully functional GCs. This is even more needed for LH, since very little mechanistic data are available despite the reorganization of mitochondrial network during the luteal phase (121). Overall, this indicates the LH signaling pathway as a modulator of the mitochondrial steroidogenic activity and dynamics.

## Ovarian Steroid Hormones and Peptides Controlling Mitochondrial Dynamics and Activity

The number and maturation of the follicles is strictly controlled by ovarian endocrine hormones (estradiol, progesterone, etc.) and intra-ovarian regulators. While the formers are synthetized by GCs and TCs, the latter include proteins secreted by oocytes (Growth Differentiation Factor 9, GDF9, bone morphogenetic protein 15, BMP15) and somatic cells (Anti-Mullerian Hormone, AMH, inhibins, activins, etc). These lasts are defined “ovarian peptides” and play an important role in ovarian aging so that they are used in evaluation of the ovarian reserve (122–125). As factors controlling the follicular atresia, we looked for reports investigating their role in regulation of mitochondria activity and dynamics (5, 126). We did not retrieve reports describing their direct role either exerted by ncRNAs. In summary, we emphazise the need to direct future research in understanding the mechanisms by which the decline in these “ovarian peptides” promote follicular atresia altering mitochondrial activity (127, 128).

Ovarian somatic cells play a major role in deciding the fate of follicles since serving other molecules essential for their growth and maintenance as estrogens and other steroid hormones. Steroids affect the mitochondrial function directly and/or indirectly: estrogens (E) and testosterone (T) are directly active in the mitochondria. Their receptors (ERs, AR), localized also in the organelle, might directly regulate DNA-encoded mitochondrial proteins (129, 130). They might exert an indirect activity affecting the nuclear transcription of proteins controlling the expression of mtDNA-encoded proteins (131).

Estrogens withdrawal characterizes the physiological and premature ovarian aging. Mitochondria are involved in synthesis of E and are also their targets. Indeed, mitochondrial function including mitochondrial bioenergetics, calcium homeostasis, and ROS-scavenger and their dynamics are regulated by E (38, 131). Indeed, glutathione peroxidase (*GPX*) and Mn-superoxide dismutase (*Mn-SOD*) are directly regulated by binding of activated *ERα* and *ERβ* to their promoters (132). Despite that, the role of estrogens as modulator of mitochondrial activity and dynamics has been scarcely addressed in ovary. The studies discussed below have been conducted mainly in other tissues and cell types and have been included since they involve molecular players non cell specific. Although some mitochondrial transcripts are directly regulated by estradiol (E_2_) like E_2_-ERs complexes (*TFAM, ATP5PB, mtATP6*, etc.), E_2_ controls their expression primarily through modulation of *NRF1* mRNA, a major transcriptional regulator of mitochondrial biogenesis (133). *ERα* and *Erβ* bind to a non-consensus ERE in the 5’ promoter of the human *NRF1*, regulating its transcription (134). It has been demonstrated that *NRF1* plays a critical role in integrating nucleus-mitochondrial interactions by initiating transcription of nuclear-encoded mtDNA-specific transcription factors including *TFAM*, *TFB1M*, and *TFB2M* (135). *NRF1* was shown to bind and increase transcription of all ten nuclear-encoded mouse *Cox* genes (136). Additional target genes of *NRF1* include *TFAM*, *TFB1M*, *TFB2M*, *SURF1*, *VDAC*, and *TOM20* genes (137–140). *TFAM, TFB1* and *TFB2* increase transcription of mtDNA and increase mitochondrial biogenesis regulating other key transcription factors including *NRF2* and *PGC-lα*. These lasts play master roles in mitobiogenesis, whereas E and ERs are key “directors” for the entire pathway. Alteration of the mitochondrial number compromises cellular metabolism and leads to an uncontrolled ROS production (141). Estradiol up-regulates the cellular levels of antioxidant enzymes (*GPX* and *Mn-SOD*). However, the mechanism by which E_2_ up-regulates those enzymes remains unidentified, but they may have implications for gender differences in lifespan (132). Estrogens withdrawal promotes a ROS scavenger activity reduction and the following ROS increase leads to a Ca^2+^ overload in the mitochondrial matrix. This last event induces the permeabilization of the inner mitochondrial membrane and the dissipation of the proton electrochemical gradient (ΔΨm) resulting in ATP depletion, further ROS production, and ultimately swelling and rupture of the organelle (142–145). Mitochondrial *ERβ* functions as a mitochondrial vulnerability factor because it is involved in ΔΨm maintenance, potentially through a mitochondrial transcription dependent mechanism (146). Furthermore, E_2_ controls mitochondrial dynamics modulating DRP1 phosphorylation *via* AKT pathways. Additionally, other proteins (MFN1, MFN2, OPA1) involved in control of mitochondrial dynamics are targets of E_2_ signaling pathways, moving from the membrane bound estrogen receptor (GPER) (147). It has been shown that GPER1 overexpression induces the levels of mitofusion 1 (*MFN1*), mitofusion 2 (*MFN2*) and Parkin (*PRKN*) mRNAs whereas mitochondrial fission 1 (*FIS1*) mRNA is reduced. Therefore, it controls mitochondrial fission/fusion and mitophagy, that is increased (148). Although E modulates the expression of several ncRNAs we did not retrieve papers highlighting their modulation as a mechanism of estrogen regulation of mitochondrial dynamics and activity.

Androgens are also steroids retrieved in the ovary and their role in follicle maturation and atresia is known since time (149). Their deficiency is likely to have a negative impact on fertility whereas their excess is a hallmark of polycystic ovary syndrome. Androgens contribute to the antral follicle formation (150). The absence of androgen receptor (AR^−/−^) induces abnormal ovarian function due to the promotion of preantral follicle growth and prevention of follicular atresia (151). The AR signaling modulates transcriptionally the mitochondrial metabolism regulating the mitochondrial pyruvate carrier (MPC). MPC inhibition results into a considerable disruption of the metabolic homeostasis, affecting ATP and antioxidant content (152). *NRF1* is also a major mediator of androgen signaling pathway. It has been shown that AR reduction led to a significant decrease in the expression of peroxisome proliferator-activated receptor γ (*PPARγ*) co-activator 1-β (*PGC1-β*) and of its downstream genes, including *NRF1* and *TFAM*. Therefore, the mitochondrial biogenesis is affected also by androgens.

In summary, estrogens and androgens protect mitochondria stimulating their ROS-scavenger activity resulting in a reduction of mitochondrial stress and mitochondrial misfolded proteins. The accumulation of the latter in the inner mitochondrial membrane space activates the unfolded protein response (UPRmt), finally initiating the mitochondrial-nuclear retrograde signaling. Noteworthy, mtUPR activates *ERα* promoting its phosphorylation by AKT whereas E_2_-ERα modulates mtUPR (54, 153–155).

## Conclusion

Despite the well-characterized role of the mitochondria in follicular maturation and atresia, less is known on how their dynamics and activity is modulated by the signaling pathways dependent on pituitary-ovarian axis hormones and how the latter influence the nucleus-mitochondrial crosstalk (**Figure 1**). The data summarized here suggest pituitary-ovarian hormones as modulator of the anterograde signaling, from nucleus to mitochondria. Among the hormones produced in the ovary, the estrogens are well-characterized regulators of mitochondrial activity and their role have been analyzed also in ovarian aging. Their absence is a marker in the ovarian aging. They are anterograde signals since their classic receptors have been localized in the mitochondria, where they modulate the expression of several genes and control different mitochondrial activity including the mtUPR. Furthermore, they modulate the expression of *NRF1* a key anterograde signal. The effects of the other ovarian steroids, such as progesterone and androgens, on ovarian aging have barely been reported. Their ability to control mitochondrial dynamics and activity in ovary has been evidenced in patients suffering from Polycystic Ovary Syndrome (PCOS), whose discussion is behind the scope of this work (156). The follicle is also site of synthesis of the so-called ovarian peptides, key modulators of ovarian aging as AMH, activins and inhibins. To our knowledge no data are available on the modulation of mitochondrial activity and dynamics by their signaling pathways.

Focusing on pituitary gonadotropins, the reported data showed a better characterization of the molecular mechanisms regulating mitochondrial dynamics and activity only for FSH that might act on anterograde signaling modulating the PINK1-Parkin pathway or *via* modulation of prohibitin level. However, these studies are not numerous. The LH signaling pathway has been far less investigated, and few papers suggested the mitochondrial dynamics is targeted by LH targets in luteal cells. Ovarian and pituitary hormones can regulate nucleus-mitochondria crosstalk also modulating the cellular content of ncRNAs involved in ovarian aging. MiR-143 is an example since it is regulated by FSH.

The molecules involved in the retrograde signaling between the two organelles, different from ATP, Acetyl-CoA and ROS among the others, are almost an unexplored during follicular maturation and atresia. Very few proteins, mitokines (humanin and FGF21), have been described as retrograde signals, which are mainly represented by ncRNAs. Despite that, it has been shown that humanin levels in follicular fluids positively associates with ovarian reserve (60). The role of the ncRNAs associated with the mitochondria (mitomiRNAs and mt-lncRNAs) has been discussed here. Given that this issue is almost unexplored in ovaries, we were surprised to find among them GAS5, a lncRNA that we and other researchers have associated with ovarian aging, which seems to be active as retrograde signal. Our unpublished data evidence that GAS5 and miR-143 have similar regulation in a context of physiological aging in testes, suggesting the need to evaluate their role as modulator of nucleus-mitochondria crosstalk in both gonads. Indeed, an imbalance of the pituitary-testes hormones occurs in physiological aging of spermatozoa and mitochondria play a major role in this process. To our surprise, in testicular somatic and germ cells they have been rarely investigated as targets of these signaling pathways but mainly as biosynthetic sites of androgens.

In conclusion, we believe that more efforts are needed to characterize the mitochondria-nucleus reciprocal crosstalk during follicle maturation and during their physiological and premature aging and how it might be targeted by the hormones active along the pituitary-ovary axis. Furthermore, considering the role that pituitary-testis hormones have on spermatozoa maturation and health.

## Perspective

Mitochondria and their crosstalk with the nucleus have been proposed as a therapeutic target for neurological diseases. Considering the role that bidirectional mitochondrial-nuclear crosstalk have in senescence and aging, we suggest it as a potential valid therapeutic target also in ovarian aging (157). Since mitochondria are main executors of the apoptosis and of the depletion mechanisms of OR, the exploitation of the molecular mechanisms underlying their regulation by the signaling pathways dependent on pituitary and ovarian hormones and peptides is needed to identify their mediators that can be targeted to preserve the ovarian health. Among them, ncRNAs, acting as anterograde and retrograde signals in nuclear-mitochondrial crosstalk, could represent an innovative therapeutic approach already suggested for several other diseases. What we consider evident is how far we are from this goal although such mediators, proteins and ncRNAs, might represent “druggable pathways” in ovarian aging.

## Author Contributions

Conceptualization, CA and MF. Investigation, MC and DC. Writing-original draft preparation, CA, MC, DC, TP, IF, and MM. Writing-review and editing, CA, MC, and DC. Visualization, MC. Supervision, CA, MM, and MF. Project administration, CA. Funding acquisition, CA. MC and DC have contributed equally to this work and share first authorship. All authors have read and agreed to the published version of the manuscript.

## Funding

This work was supported by: The Italian Workers’ Compensation Authority 571 (grant no 12010), Sensor Regione Campania (grant no 23), Goodwater Regione 572 Campania (POR Campania FESR 2014/2020 O.S. 1.1 Az. 1.1.3 E 1.1.4—CUP 573 B63D18000150007), POR FESR 2014-2020- Projects (RARE PLATNET, SATIN and 574 COEPICA) Regione Campania and “Legge Regionale 5”- Annualità 2008.

## Conflict of Interest

The authors declare that the research was conducted in the absence of any commercial or financial relationships that could be construed as a potential conflict of interest.

## Publisher’s Note

All claims expressed in this article are solely those of the authors and do not necessarily represent those of their affiliated organizations, or those of the publisher, the editors and the reviewers. Any product that may be evaluated in this article, or claim that may be made by its manufacturer, is not guaranteed or endorsed by the publisher.
